# The Use of Platelet-Rich Plasma for the Treatment of Osteonecrosis of the Femoral Head: A Systematic Review

**DOI:** 10.1155/2020/2642439

**Published:** 2020-03-07

**Authors:** Jun Han, Fuqiang Gao, Yajia Li, Jinhui Ma, Wei Sun, Lijun Shi, Xinjie Wu, Tengqi Li

**Affiliations:** ^1^Department of Orthopedic Surgery, Peking University China-Japan Friendship Hospital School of Clinical Medicine, China; ^2^Centre for Osteonecrosis and Joint-Preserving & Reconstruction, Department of Orthopedic Surgery, Beijing Key Laboratory of Arthritic and Rheumatic Diseases, China-Japan Friendship Hospital, Peking Union Medical College, National Health and Family Planning Commission of the People's Republic of China, China; ^3^Department of Dermatology, Xiangya Hospital, Central South University, China; ^4^Department of Orthopedic Surgery, China-Japan Friendship Institute of Clinical Medicine, Peking Union Medical College, China

## Abstract

**Background:**

As a pathological process, osteonecrosis of the femoral head (ONFH) is characterized by the avascularity of the femoral head, cellular necrosis, microfracture, and the collapse of the articular surface. Currently, critical treatment for early-stage ONFH is limited to core decompression. However, the efficacy of core decompression remains controversial. To improve the core decompression efficacy, regenerative techniques such as the use of platelet-rich plasma (PRP) were proposed for early-stage ONFH. As a type of autologous plasma containing concentrations of platelets greater than the baseline, PRP plays an important role in tissue repair, regeneration, and the differentiation of mesenchymal stem cells (MSCs). In this review, we present a comprehensive overview of the operation modes, mechanism, and efficacy of PRP for early-stage ONFH treatment.

**Methods:**

We searched for relevant studies in the PubMed, Web of Science, and Embase databases. By searching these electronic databases, the identification of either clinical or experimental studies evaluating PRP, MSC, core decompression, and ONFH was our goal.

**Results:**

Seventeen studies of PRP and avascular necrosis of the femoral head were evaluated in our review. Ten studies related to the possible mechanism of PRP for treating ONFH were reviewed. Seven studies of the operation modes of PRP in treating ONFH were identified. We reviewed the efficacy of PRP in treating ONFH systematically and made an attempt to compare the PRP operation modes in 7 studies and other operation modes in past studies for early-stage ONFH treatment.

**Conclusion:**

PRP treats ONFH mainly through three mechanisms: inducing angiogenesis and osteogenesis to accelerate bone healing, inhibiting inflammatory reactions in necrotic lesions, and preventing apoptosis induced by glucocorticoids. In addition, as an adjunctive therapy for core decompression, the use of PRP is recommended to improve the treatment of early-stage ONFH patients, especially when combined with stem cells and bone grafts, by inducing osteogenic activity and stimulating the differentiation of stem cells in necrotic lesions.

## 1. Introduction

As a pathological process, osteonecrosis of the femoral head (ONFH) is characterized by avascularity of the femoral head, cellular necrosis, microfracture, and collapse of the articular surface [1–3]. Presently, the pathological mechanism underlying ONFH has not been explained clearly, but the use of glucocorticoids and alcohol is recognized as the most common pathogenic factor [4, 5]. The prevalence of ONFH worldwide is unknown. In China, the overall number of osteonecrosis cases has reached 7 million, and 100,000 to 200,000 new cases of ONFH are diagnosed each year [6]. Meanwhile, it is estimated that 15,000–20,000 new cases of ONFH are diagnosed in the US each year [7]. The incidence of ONFH is increasing, and more patients with ONFH are now diagnosed in the clinic [8, 9].

At present, total hip arthroplasty (THA) remains the most widespread procedure used to treat end-stage ONFH [10]. However, arthroplasty is not a suitable option for patients who are young or with early-stage ONFH because THA reduces the quality of life of patients by restricting the motion of the hip and resulting in some complications. Therefore, young patients who suffer early-stage ONFH tend to choose conservative treatment to avoid or at least delay THA. Currently, core decompression of the hip is the most common conservative treatment for early-stage ONFH, but its cure rate is only 63.5%, and its efficacy remains controversial [11–13]. This is because core decompression is used to decompress the intraosseous pressure and remove necrotic tissue from the hip, but it does not have superior effects on facilitating osteoanagenesis in the necrotic area [14, 15]. For this reason, with the purpose of improving the results of core decompression, regenerative techniques such as the use of platelet-rich plasma (PRP) have been proposed to address early-stage ONFH [16].

As a specimen of autologous plasma containing platelets at concentrations above the baseline [17], PRP contains growth factors in addition to platelets, such as platelet-derived growth factor (PDGF), transforming growth factor-*β* (TGF-*β*), basic fibroblast growth factor (bFGF), endothelial growth factor (EGF), and vascular endothelial growth factor (VEGF), which have an important effect on tissue repair and the proliferation and differentiation of mesenchymal stem cells (MSCs) [16, 18]. The application of PRP in orthopaedics began earlier this decade and has been combined with bone grafts to augment vertebral fusion and fracture treatment [19]. van der Jagt et al. reported that the use of PRP could relieve pain and halt the disease progression of patients with ONFH [20]. Currently, increasing experimental and clinical evidence has shown that PRP may play an effective role in ONFH treatment [21, 22].

In this review, we present a comprehensive overview concerning the operation modes and mechanism of PRP for early-stage ONFH treatment. Then, we present a systemic review of the comparison of PRP with other therapies for treating early-stage ONFH.

## 2. Materials and Methods

This systemic review was conceived in accordance with the Preferred Reporting Items for Systematic Reviews and Meta-Analyses (PRISMA) reporting guidelines for the conduct of systematic reviews and meta analyses of intervention trials (Additional File 1). The review was not prospectively registered because it did not meet the criteria for registration with PROSPERO. Ethical approval of this study was not necessary because it was a systematic review of the accessible literature and any individual patient data were not involved.

### 2.1. Search Strategy

Three electronic databases (PubMed, Embase, and Web of Science) were searched, and we used terms and Boolean operators as follows: “(platelet-rich plasma) AND (avascular necrosis OR aseptic necrosis OR osteonecrosis OR necrosis) AND (femoral head OR hip)”. The search strategies are found in Additional File 2. We did not limit the year of publication, publication status, or language, and there were also no limitations in terms of any particular study design, including randomized or unrandomized control trials, cohort studies, and case reports. Moreover, the references of the articles were also checked manually to identify other potentially relevant literature. We did not search unpublished articles.

### 2.2. Eligibility Criteria and Study Selection

The studies that met the following criteria were considered eligible: the study enrolled patients with ONFH and the study researched PRP and ONFH. Letters, comments, editorials, and practice guidelines were excluded. Then, the studies were selected independently by 2 of the authors, and any differences in opinion were resolved through discussion with mediation from a third peer if needed (LS, XW, and TL). All information on the included studies is listed in Additional File 2.

### 2.3. Data Extraction and Quality Assessment

All potential data were extracted and assessed by two reviewers (LS and XW) independently. Discrepancies between the two collaborators were discussed to reach consensus. Data were extracted with respect to participant characteristics, authors, journal, publication date, study design, operation technique, and outcomes for each study included in our review. Participant information included the number, age, and stage of the participants. Information about outcomes was recorded, including scores of pain and function, MRI and X-ray images, conversion to THA, and collapse of the hip. The quality of each of the included articles was assessed using the guidelines of the U.S. Preventive Services Task Force by two reviewers (LS and XW).

### 2.4. Statistical Analysis

We first provided a comprehensive overview of the mechanism underlying PRP and its relationship with ONFH treatment in recent years. Then, the data from studies about PRP clinical application were collected, and we categorized the clinical operation modes of PRP into several groups and compared their therapeutic effects with those of other therapies for ONFH treatment systematically. RevMan v5.3 was used to analyze data extracted from included articles. Continuous outcomes were addressed as the mean ± standard deviation. Heterogeneity of articles was estimated by the *I*^2^ statistics; substantial heterogeneity was reflected by *I*^2^ > 50%. *P* < .05 was considered statistically significant.

## 3. Results

**Figure 1:**
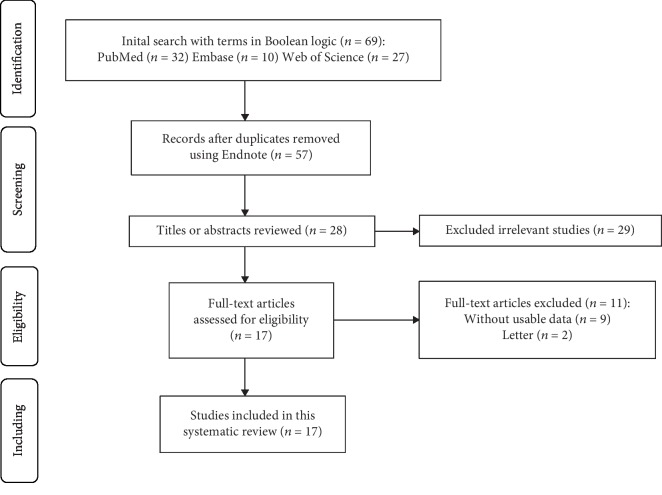
The selection process of this review.

A total of 69 studies were initially searched within the PubMed, Embase, and Web of Science databases (Figure 1). After the removal of duplicates, title screening, and abstract or full-text screening, 17 studies that researched PRP and ONFH were identified in this review; 10 studies investigated the PRP mechanism, and 7 studies addressed PRP clinical applications. We reviewed the mechanism of PRP and its role in the treatment of ONFH. Then, we systematically reviewed the efficacy of PRP in treating ONFH and attempted to make a comparison of PRP operation modes and other operation modes in past studies used in early-stage ONFH treatment. Data could not be pooled because of the methodological heterogeneity and limited number of the available controlled studies.

### 3.1. The Mechanism of PRP in ONFH Treatment

#### 3.1.1. PRP Accelerates Angiogenesis and Osteogenesis in ONFH

**Figure 2:**
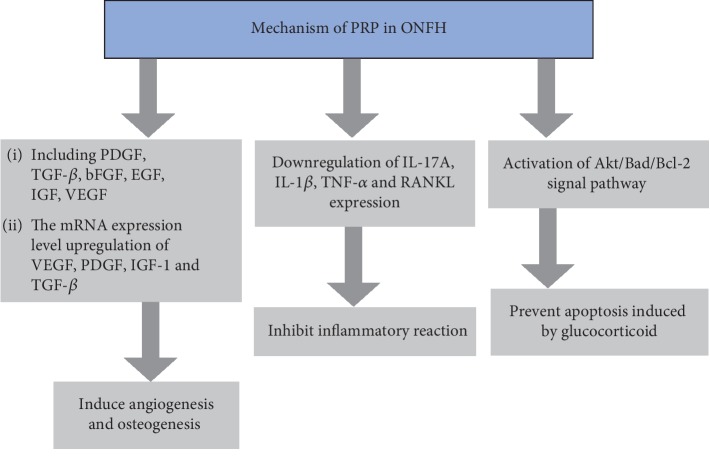
The mechanism of PRP in ONFH treatment. PRP: platelet-rich plasma; PDGF: platelet-derived growth factor; TGF-*β*: transforming growth factor-*β*; bFGF: basic fibroblast growth factor; EGF: endothelial growth factor; IGF: insulin-like growth factor; VEGF: vascular endothelial growth factor; IL-17A: interleukin-17A; IL-l*β*: interleukin-1*β*; IL-6: interleukin-6; TNF-*α*: tumor necrosis factor-*α*; RANKL: receptor activator of nuclear factor-*κ* B ligand; Akt: protein kinase B; Bad: Bcl-2-associated death promoter; Bcl-2: B-cell lymphoma 2.

After the use of PRP was verified as a useful management strategy for ONFH treatment by Yokota et al. [23], many clinical and epidemiological studies started to research the mechanism of PRP in ONFH treatment. Numerous studies have reported that enhancing osteogenesis and angiogenesis to reconstruct the bone structure of necrotic areas is the main mechanism involved in early-stage ONFH treatment [1, 24, 25]. In light of the above rationale, PRP could augment core decompression and bone graft substitutes to treat early-stage ONFH through increasing the levels of various cytokines that initiate and regulate proliferation, differentiation, and angiogenesis [16, 17, 26] (Figure 2). The cytokines identified in PRP include platelet-derived growth factor (PDGF), transforming growth factor- (TGF-) *β*, basic fibroblast growth factor (bFGF), endothelial growth factor (EGF), insulin-like growth factor (IGF), and vascular endothelial growth factor (VEGF), some of which have been examined efficiently by many researchers. Nakamae et al. reported that combining FGF-2 administration with vascular bundle implantation may enhance the treatment of avascular necrosis in the hips of rabbits [27]. Suzuki et al. reported that VEGF administration increased surgically induced angiogenesis and neovascularization in necrotic bone 1 week after arteriovenous bundle implantation [28]. Yang et al. found that VEGF gene transfection for the treatment of ONFH in rabbits increased bone formation compared to that in the control group, which did not receive VEGF transfection therapy [24]. Several articles have indicated that each growth factor individually induces angiogenesis and osteogenesis. In contrast, some articles reported growth factors that contributed the suppression of osteoanagenesis; the single high-dose administration of TGF-*β* (335 *μ*g into a humeral canine model) or VEGF (0.5 *μ*g into a rat bone defect) suppressed bone regeneration [29, 30]. Therefore, angiogenesis is a complex process requiring a finely tuned balance between numerous growth factors. Brill et al. reported that the concomitant effect of VEGF and bFGF on the vessel formation process seemed to be more essential than a single VEGF or bFGF administration alone [31]. Thus, PRP could create an optimal environment to increase the repair capacity of ONFH by regulating the interactions of numerous growth factors with different systems.

With the exception of PRP itself, which contains angiogenic factors, Tong et al. reported that the mRNA expression levels of VEGF, PDGF, IGF-1, and TGF-*β* in ONFH mice were upregulated tremendously after PRP treatment compared to those in the control group (PBS-treated). PRP may increase the production of angiogenic factors via upregulation of the angiogenic gene pathway, which may contribute to angiogenesis in femoral head osteonecrosis [32].

#### 3.1.2. PRP Treatment Inhibits the Inflammatory Reaction in ONFH

ONFH is associated with synovitis, which is characterized by the presence of inflammatory cells and proinflammatory cytokines in necrotic lesions and synovium [33, 34]. Previous research has found that the synovium is saturated by CD4+ T cells, macrophages, and some CD8+ T cells during ONFH pathogenesis [35]. Some proinflammatory cytokines, such as interleukin-1*β* (IL-1*β*), IL-6, TNF-*α* (tumor necrosis factor-*α*), and IL-17, are also involved in the pathophysiological process of ONFH [36, 37]. Although the mechanism of ONFH induction is not fully understood, inflammatory cytokines have been reported to be the critical mediators of ONFH pathogenesis according to previous studies [38, 39]. Zou et al. reported a typical positive association between IL-17 and pain severity in ONFH [36].

Tong et al. reported that the mRNA expression and concentrations of inflammatory cytokines, such as IL-1*β*, TNF-*α*, IL-17A and receptor activator of nuclear factor-*κ* B ligand (RANKL), were significantly downregulated in the PRP treatment group compared to the control group in synovial cells in a ONFH mouse model [32]. This result indicated that PRP treatment effectively suppressed the expression of IL-17A, IL-1*β*, TNF-*α*, and RANKL in ONFH. Interestingly, this study also suggested that PRP treatment upregulated the mRNA expression levels and concentrations of interferon- (IFN-) *γ* and IL-6 compared with those in synovial cells in the control group. This result indicated that PRP treatment increased IFN-*γ* and IL-6 levels in ONFH, which is in contradiction with the increase in IL-6 in the local synovium in ONFH and the negative effects of IL-6 on osteoblast differentiation [34, 40]. With regard to the downregulation of IL-6 and IFN-*γ*, Tong et al. proposed that PRP treatment could promote chondrogenic proliferation and differentiation, which contribute to the production of IL-6 and IFN-*γ* in the maintenance of the articular cartilage [41, 42].

Overall, PRP treatment downregulates the inflammatory reaction, which may stop ONFH progression and alleviate pain by interrupting the process of inflammatory damage in the joint.

#### 3.1.3. PRP Exosome Treatment Prevents Apoptosis Induced by Glucocorticoids in ONFH

Glucocorticoid use can cause ONFH by inducing cell apoptosis, which accounts for bone loss and angiogenesis impairment [43, 44]. Glucocorticoids modulate endoplasmic reticulum (ER) stress to induce cell apoptosis. Therefore, if a treatment could prevent cell apoptosis by mediating ER apoptosis, it could reverse the progression of ONFH.

Among the three major signal transduction pathways involved in ER stress, Tao et al. found that the protein kinase R-like endoplasmic reticulum kinase (PERK) pathway is closely associated with apoptosis [45]. Glucocorticoids activate PERK by phosphorylation of eukaryotic translation initiation factor 2*α* (eIF2*α*). As a downstream protein affected by PERK, CCAAT-enhancer-binding protein homologous protein (CHOP) suppressed B-cell lymphoma 2 (Bcl-2) protein expression, after which caspase-3 was cleaved, contributing to cell apoptosis [46, 47].

Tao et al. reported that ER stress-induced apoptosis could be inhibited by PRP exosomes without altering PERK activation and CHOP expression [45]. After adding PRP exosomes to BMSCs (bone mesenchymal stem cells) along with dexamethasone, Akt (protein kinase B) and Bad (Bcl-2-associated death promoter) were phosphorylated, Bcl-2 expression was increased, and cleaved caspase-3 could not be detected. Therefore, Bcl-2 might be a key protein involved in the antiapoptotic effects of PRP exosomes. PRP exosomes inhibited cell apoptosis mainly through the activation of the Akt/Bad/Bcl-2 signalling pathway.

In addition to inhibiting apoptosis induced by dexamethasone, PRP treatment could also reduce the toxic effects of dexamethasone directly. Tong et al. found that a reduction in the serological levels of antiglucocorticoid IgG occurred in PRP-treated ONFH mice compared with the control group, which indicated that the humoural glucocorticoid concentration was decreased by PRP [32].

### 3.2. The Applications of PRP in ONFH Treatment

#### 3.2.1. Combination of PRP with Stem Cells to Treat ONFH

Houdek et al. conducted a prospective study based on 22 early-stage ONFH participants in the Mayo Clinic, who were treated with PRP combined with bone marrow-derived mesenchymal stem cells (BmMSCs) after core decompression, and observed that more than 93% of the patients did not suffer from femoral head collapse, and 84% of the patients did not exhibit conversion to THA at a 3-year follow up (Table 1). Additionally, this study demonstrated that the mean modified Kerboul angle of the necrotic lesions improved from 205° ± 47° to 172° ± 48° according to a 12-month postoperative MRI assessment, which indicated a reduced risk of progressing to collapse [48, 49]. MSCs are capable of enhancing tissue regeneration by differentiating into various mesenchymal phenotypes, such as [50] osteoblasts, chondrocytes, and adipocytes. When MSCs are combined with PRP to treat ONFH, the necessary growth factors in PRP could promote the potential of MSCs to differentiate into new bone and blood vessels. Thus, PRP enhances the osteoanagenic effect of MSCs to improve the healing of early-stage ONFH.

In addition to BmMSCs, PRP combined with adipose-derived MSCs (AdMSCs) has also been used for ONFH treatment. Due to the limited number of surgical cases receiving treatment with PRP and AdMSCs, none of the studies utilizing large sample volumes has systematically evaluated its therapeutic effects thus far. Pak published two case reports for 4 patients with stage IV ONFH who received treatment by the injection of a PRP/AdMSC mixture into the femoral head under ultrasound guidance, which showed the long-term improvement in pain and motion range and the regeneration of bone by MRI for at least 7 to 12 months [51, 52]. In another case report from Pak et al. [53], one patient with stage I ONFH received PRP/AdMSC mixture treatment once and PRP treatment every week for 4 weeks for subsequent treatment. Eventually, this patient displayed the complete resolution of hip pain and motion function abnormalities at a 21-month follow-up, and MRI showed significant signal changes in both the T1 and T2 views of the femoral head before and after PRP/AdMSC treatment. This is the first complete resolution of ONFH via treatment with the injection of PRP/AdMSCs percutaneously.

The combination of AdMSCs and PRP may produce improved therapeutic effects for ONFH than BmMSC/PRP treatment. The concentration of AdMSCs (approximately 200,000/g) was higher than that of BmMSCs (approximately 6000-60,000/g) [53], and the source of BmMSCs was restricted by certain diseases, such as osteoporosis and leucocythemia [54]. The study from Pak et al. also supported the use of AdMSCs, suggesting that AdMSCs show greater potency in promoting regeneration and osteogenic differentiation than BmMSCs in the treatment of osteonecrosis [53].

#### 3.2.2. PRP Combined with Synthetic or Autologous Bone Grafts for the Treatment of ONFH after Core Decompression

Currently, there are only three articles that have reported the use of PRP combined with bone grafts to treat ONFH during core decompression surgery. The three articles all showed significant outcomes for the treatment of ONFH, particularly for early-stage patients.

Guadilla et al. enrolled one patient with Grade IIA ONFH and three patients with Grade IIB ONFH who were treated with PRP and autologous bone grafting after core decompression through arthroscopy. The four patients all exhibited a significant reduction in the visual analogue score (VAS) and were restored to a normal life state in the fifth month. A significant improvement in symptoms was observed during the early follow-up process, and representative bone formation was observed by MRI soon afterwards. Considering the limits in terms of the patient number and the lack of advanced ONFH patients, this study cannot provide sufficient evidence to support PRP treatment [55]. Samy conducted a prospective study of 40 hips (30 patients) in modified Ficat stages IIb and III ONFH that were treated with an operation procedure consisting of core decompression, a composite iliac bone graft, and PRP [56]. Significant pain relief was reported in 34 hips (85%), and the Harris hip score (HHS) was improved from 46.0 ± 7.8 preoperatively to 90.28 ± 19 postoperatively at a 4-year follow-up. This result was much better than that of a study without PRP reported by Mont et al., who used the technique of core decompression and iliac bone graft [57]. D'Ambrosi et al. evaluated 24 hips in 16 patients, which included all Ficat classification stages, treated by core decompression, the injection of PRP and MSCs, and synthetic bone grafts [58]. Clinical failure was defined as receiving THA by 75 months of follow-up. The total survivorship of this operation is 50%. The survival rate was 80% for patients in stage I+II and 28.6% for patients in stage III+IV. In comparison with stage I and II patients, the patients in stages III and IV suffered a higher rate of failure for the operation.

### 3.3. Effectiveness of PRP in comparison to Other Treatments in Treating ONFH

Currently, the use of PRP to treat ONFH is just beginning to be utilized, and there are few articles describing the study of PRP efficacy in treating ONFH. The total number of articles that could be retrieved included 4 case reports and 3 self-controlled studies. Moreover, the main function of PRP is as an adjuvant substitute for core decompression to treat ONFH. For this reason, we reviewed articles to make a comparison between previous modes of core decompression and the use of PRP in combination with core decompression.

To improve bone regeneration, the application of core decompression in association with stem cells is an appealing possibility. Hernigou and his colleagues are the pioneers in applying stem cells to ONFH treatment. They conducted a prospective study of 189 hips in 116 patients receiving core decompression and autologous BmMSC grafts. At the 5- to 10-year follow-up, THA was necessary for 9 of 145 hips with early-stage ONFH (Steinberg stage I and stage II), which indicated a high 95% success rate [59]. However, the Hernigou study defined Steinberg stage II as a hip with an abnormal MRI scan and an abnormal radiograph in a patient presenting with sclerosis or cystic lesions within the femoral head but without a crescent line, which was equivalent to the stage in between the actual Steinberg stage I and II classifications [60]. Thus, the strict selection of early patients contributed to the high success rate. Several studies discovered that the survivorship rate free from collapse for early-stage patients who received core decompression combined with MSC was 85%~90% 24~60 months after follow-up [61, 62]. All the aforementioned studies agree that core decompression associated with stem cells can improve postoperative outcomes compared to core decompression alone. What would be the results from combining PRP with core decompression and stem cells? Houdek et al. applied PRP in combination with BmMSCs and core decompression to the treatment of early-stage ONFH in 31 hips in 22 patients (Steinberg stage I and stage II), which revealed that more than 93% and 84% of patients did not progress to femoral head collapse and received THA 36 months after follow-up, respectively [48]. Houdek et al. also analyzed the BmMSC content of bone marrow concentrate (BMC) by testing the nucleated cell quantity and colony-forming units (CFUs), which revealed that patients who received a higher mean number of CFUs and a higher mean concentration of nucleated cells per millilitre of BMCs were more likely to not need repeated surgical procedures (THA or decompression). In addition, this study showed that 80% of patients classified as high risk (modified Kerboul Grades 3 and 4) received repeated surgical procedures (THA or core decompression), while none of low-risk (modified Kerboul Grades 1 and 2) patients suffered hip collapse [48, 49].

By simply comparing the outcomes between the studies by Hernigou et al. and Houdek et al., it was revealed that for preserving femoral head integrity, the curative effect of combining PRP with BmMSCs and core decompression was similar to that of combining BmMSCs and core decompression for treating early-stage ONFH. However, when considering the strict selection of patients in the Hernigou et al. study, which excluded patients with crescent signs, PRP indeed could augment the curative effects of combining BmMSCs and core decompression for early-stage ONFH.

By considering the use of synthetic or autologous bone grafts to augment core decompression, Rosenwasser et al. revealed a significant clinical hip protection rate (85%) in 15 hips in 13 patients with stages II and III ONFH during 10-15 years of follow-up [63]. Wang et al. evaluated 110 patients (138 hips) with stage ARCO IIA–IIIA ONFH treated with core decompression combined with a mixture of autologous bone and demineralized bone matrix. The definition of clinical failure was a Harris hip score below 80 points or the necessity for further surgical procedures (THA or osteotomy). In conclusion, the study reported an 85% survivorship rate in stages IIA and IIB during 25 months of follow-up [64]. However, some studies have produced the exact opposite results for core decompression combined with bone graft. Yu et al. enrolled 18 patients with 19 hips (stage IIC: 6 hips, stage IIIA: 13 hips) who were treated with core decompression combined with synthetic bone grafts. The definition of clinical failure was conversion to THA or the progression of head collapse. In the end, 3 stage IIC hips and 8 stage IIIA hips were converted to total hip replacement at a mean time point of 8.5 months postoperatively. The other six hips progressed to collapse of the femoral head. Only two hips (10.5%) were free of progression to collapse during long-term follow-up (5 years) [65]. The above results suggest that the efficacy of synthetic or autologous bone grafts combined with core decompression has been debatable so far. According to the published articles, the use of a mixture of PRP and autologous bone in the core decompression track showed improved results versus the results of grafting bone alone. Samy performed an operation procedure using core decompression, composite iliac bone grafts, and PRP on 30 ONFH patients with 16 stage IIb hips and 24 stage III hips, which were graded according to the modified Ficat classification, and 90% of patients were free from THA at 4 years of follow-up [56]. Guadilla et al. treated 4 patients with Grade IIA and IIB ONFH with PRP and autologous bone grafting after core decompression, which showed that cartilage integrity in the 4 patients was preserved for an average of 14 months of follow-up [55].

These results suggested that bone grafting combined with a mixture of PRP and MSCs could theoretically have effects on improving the success rate of decompression. Bone grafting provides provisional mechanical support after removing the necrotic lesion, while PRP stimulates the differentiation of MSCs and plays a crucial role in osteogenesis and angiogenesis to induce bone healing. However, similar to the results of other studies of core decompression [66–68], PRP was not shown to be an effective method for treating advanced stage ONFH. D'Ambrosi examined 24 hips in 16 patients from all Ficat classification stages that were treated by core decompression and an injection of a mixture of synthetic bone, PRP, and MSCs. The survival rate was 80% in early-stage patients; however, only 28.6% of patients in the advanced stage survived the collapse [58]. Therefore, for treating early-stage ONFH, the outcomes of core decompression may be improved by combining PRP with other regenerative therapies, but PRP is not suitable for advanced stage ONFH or necrotic lesions larger than 50% [69]. This was verified by the research conducted by Yoon et al., which determined the importance of both the Ficat stage and lesion size in ONFH treatment [70].

## 4. Conclusion

Currently, PRP is a possible treatment for various medical problems that may stimulate and accelerate soft-tissue healing and regeneration [19, 71]. In particular, in the treatment of bone nonunion and diabetic neuropathic foot ulcers, many clinical studies have produced satisfactory results or results that are even better than the standard of care [72–74]. However, for the treatment of ONFH, PRP alone will not work very well to treat ONFH. Because ONFH is characterized by osteocyte apoptosis as a consequence of the interruption of the blood supply, the most important steps for treating ONFH are facilitating osteogenesis and angiogenesis and restoring bone formation to reconstruct the support at the joint surface. PRP must be administered in association with core decompression and other treatments to play its role.

In summary, PRP could not reverse the pathophysiological course of ONFH; however, PRP is recommended as an adjunctive therapy for core decompression combined with stem cells and bone grafts (autologous or allogeneic) to induce osteogenic activity and stimulate the differentiation of stem cells in ARCO stage I and II patients (the Association of Research of Osseous Circulation) [58]. PRP works mainly through three mechanisms: inducing angiogenesis and osteogenesis to accelerate bone healing, inhibiting inflammatory reactions in necrotic lesions, and preventing apoptosis induced by glucocorticoids. The use of PRP in combination with other techniques may show potent efficacy in treating ONFH, and this treatment method may become more popular. However, further prospective randomized clinical trials of PRP are needed to determine its efficacy; the use of a standardized preparation and the ratio of stem cells used for the therapeutic algorithm must be investigated in the future.

In general, our review also showed various limitations that should not be ignored. First, the number of included studies that could be found was relatively small. In total, 7 studies evaluated the efficacy of different operation modes of PRP; 3 articles described prospective studies, and the other 4 studies described case reports, all of which showed a low level of evidence-based medical classification. Second, there were no uniform standards for the operation procedure and the curative effect evaluation of PRP among the articles, which resulted in high heterogeneity in this study. In addition, there may be publication biases that affect every included article reporting good results.

## Figures and Tables

**Table 1:** Detailed description of the 7 studies about PRP clinical application in the systematic review.

Author	Level of evidence	Patient/hip treated	Age (years)	Staging	Technique	Follow-up	Hip survivorship (%)
Houdek et al. [48]	Level II prospective descriptive study	22/35	43	Steinberg stage I, II	PRP/BmMSCs/core decompression	36 months	90%
Pak [52]	Level V case report	2/2	29, 47	Stage IV	PRP/AdMSC mixture under ultrasound guidance	3 months	100%
Pak [51]	Level V case report	2/2	34, 39	Stage IV	PRP/AdMSC mixture under ultrasound guidance	16 months	100%
Pak et al. [53]	Level V case report	1/1	43	Ficat classification stage I	PRP/AdMSC mixture under ultrasound guidance	21 months	100%
Guadilla et al. [55]	Level V case report	4/4	/	Steinberg stage IIa, IIb	PRP/autologous bone grafting/core decompression through arthroscopy	14 months	100%
Samy [56]	Level II prospective descriptive study	30/40	36.7	Modified Ficat classification stages IIb and III	PRP/autologous bone grafting/core decompression	41.4 months	90%
D'Ambrosi et al. [58]	Level II prospective descriptive study	16/24	42	All the Ficat classification	PRP/MSCs/synthetic bone graft/core decompression	75 months	50% (80% for early patients and 28.6% for late patients)

PRP: platelet-rich plasma; BmMSCs: bone marrow-derived mesenchymal stem cells; AdMSCs: adipose-derived MSCs.

## Abbreviations

PRP: Platelet-rich plasma
ONFH: Osteonecrosis of the femoral head
MSCs: Mesenchymal stem cells
BmMSCs: Bone marrow-derived mesenchymal stem cells
AdMSCs: Adipose-derived mesenchymal stem cells
HHS: Harris hip score
THA: Total hip arthroplasty
PDGF: Platelet-derived growth factor
TGF-*β*: Transforming growth factor-*β*
bFGF: Basic fibroblast growth factor
EGF: Endothelial growth factor
IGF: Insulin-like growth factor
VEGF: Vascular endothelial growth factor
IL-17A: Interleukin-17A
IL-l*β*: Interleukin-1*β*
IL-6: Interleukin-6
TNF-*α*: Tumor necrosis factor-*α*
ER: Endoplasmic reticulum
PERK: Protein kinase R-like endoplasmic reticulum kinase
eIF2*α*: Eukaryotic translation initiation factor 2*α*
RANKL: Receptor activator of nuclear factor-*κ* B ligand
Akt: Protein kinase B
Bad: Bcl-2-associated death promoter
Bcl-2: B-cell lymphoma 2
CHOP: CCAAT-enhancer-binding protein homologous protein
VAS: Visual analogue score
BMC: Bone marrow concentrate
CFUs: Colony-forming units
ARCO: Association Research Circulation Osseous.
